# A Fully Biological Gas-Exchange Membrane toward a Biofabricated, Booster Lung

**DOI:** 10.1021/acsbiomaterials.6c00046

**Published:** 2026-04-13

**Authors:** Erica M. Comber, Kalliope G. Roberts, Isabel M. Joyce, Rachelle N. Palchesko, Daniel J. Shiwarksi, Xi Ren, Adam W. Feinberg, Keith E. Cook

**Affiliations:** † Department of Biomedical Engineering, 6612 Carnegie Mellon University, Pittsburgh, Pennsylvania 15213, United States of America; ‡ Department of Materials Science & Engineering, Carnegie Mellon University, Pittsburgh, Pennsylvania 15213, United States of America; § Department of Bioengineering, 6614 University of Pittsburgh, Pittsburgh, Pennsylvania 15213, United States of America

**Keywords:** biofabrication, tissue engineering, collagen I, artificial lung, booster lung, gas exchange

## Abstract

A means of long-term respiratory support is needed for the nearly one million chronic lung disease patients hospitalized annually. Extracorporeal membrane oxygenation can support patients for months, but clot formation within oxygenators and bleeding complications make it infeasible for permanent support. An endothelial cell coating on these devices could leverage cells’ ability to reduce clot initiation and propagation, but long-term binding to artificial materials has not been achieved. The goal of these studies was to engineer a preliminary, fully biological tissue that mimics the alveolar-capillary barrier and could function as the gas-exchange membrane of an implantable, biofabricated support lung for years. High-concentration, type I collagen membranes were made to be 18.8 ± 3.6 μm-thick and characterized in terms of mechanical strength, water permeability, and oxygen transfer under static, air–liquid conditions. The membranes were cocultured with human umbilical vein endothelial cells (HUVECs) and A549 lung epithelial cells on opposing sides to evaluate tissue viability in air–liquid conditions and permeability to the albumin mimic, 70 kDa-FITC dextran. The 18.8 ± 3.6 μm-thick acellular collagen I hydrogel withstood ≥120 mmHg and transferred 2.16 ± 0.5 μL/cm^2^/mmHg/h of plasma. It was oxygen permeable and produced 75% of the gas transfer of a 51 μm, implantable, silicone sheet. Cell cocultures remained viable in air–liquid conditions, and dextran permeability emphasized the need to include an alveolar epithelium to improve barrier function. Lastly, the method was expanded into casting a parallel-plate, perfusable channel as the functional, representative element of a booster lung. The channel provided a 16.8 ± 3.4 μm diffusion distance across 4 cm^2^ of surface area and maintained an air–liquid interface. Future work should examine cross-linking the collagen I for equally strong but thinner membranes (≤10 μm), transition to induced pluripotent stem cell cultures, and implement multichannel casting to increase the surface area needed for a biofabricated, intracorporeal, support lung.

## Graphical abstract

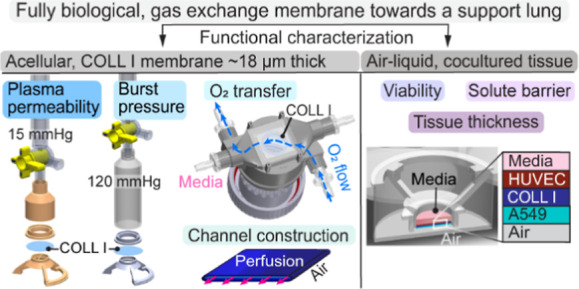

## 1 Introduction

Chronic lung disease is a major global health problem, and in the U.S. afflicts more than 16 million people and causes ∼140,000 deaths per year.^1–4^ The only treatment that can restore lung function in patients with end-stage organ failure is transplantation, but limited donor organs result in only 3000 transplants annually, and 5 year mortality post-transplantation exceeds 40%.^5^ Medical devices utilized for extracorporeal membrane oxygenation (ECMO) can support end-stage patients for a few weeks to months, but prolonged support is rare due to poor circuit hemocompatibility. The synthetic, blood-contacting materials used in ECMO circuits, particularly hollow fiber membrane oxygenators, provide nucleation sites for the activation of the coagulation cascade. Clot accumulates and causes functional failure of the device within 1–4 weeks.^6–9^ Systemic anticoagulants slow clotting, but doses must be limited to mitigate adverse bleeding events.^10^ Local anticoagulation of synthetic blood-contacting surfaces is a potential solution, but to date, heparin and other surface coatings have proven to be effective only over short periods (<6 h).^11^

The native alveolar capillary barrier is the only gas-exchange interface that functions for several years, and its longevity is driven largely by its anticoagulant endothelial cell lining. This lining has accordingly been investigated within ECMO oxygenators.^12–16^ However, the synthetic materials within oxygenators lack integrins for direct cell adhesion and require a coating of ECM proteins which can delaminate or break down over time.^17,18^ Despite the nanoscale protein coating, cells also sense the underlying substrate stiffness, which is several orders of magnitude greater than the native environment typical for a healthy cell phenotype.^19–21^ Thus, endothelial cell adherence to gas permeable, synthetic surfaces has lasted for, at most, one month under dynamic cell culture.^17,22–30^

A booster lung constructed from endothelialized extracellular matrix materials could result in a more biocompatible means of gas exchange capable of providing years of respiratory support. However, a natural membrane has not yet been constructed from solutions of ECM protein that is mechanically stable, thin enough for oxygen diffusion, and permeable to liquid and nutrients at rates that support and maintain viable tissue under air–liquid conditions.^31,32^ Here, we bioengineered a first-generation, fully biological gas-exchange membrane consisting of a thin ECM scaffold cellularized with endothelial and epithelial cells and evaluated it with respect to functional metrics. This work built upon an approach to fabricate strong, thin, collagen type I (COLL I) sheets via dehydration and measured the transport of oxygen, water from plasma, and albumin-sized molecules. The membranes were then cellularized with human umbilical vein endothelial cells (HUVECs) on one side and A549 lung epithelial cells on the other side. This matches endothelial and epithelial cell types previously cocultured on synthetic materials under air–liquid interfaces and thus offers an early, comparable measure of cell function prior to studies with primary or iPSC-derived cells.^33^ The viability and 3D structure of the membrane were assessed using confocal and multiphoton imaging, and 70 kDa dextran permeability was measured to characterize barrier function capabilities. Lastly, the method was expanded to form a perfusable, parallel-plate channel, representing a potential blood–gas pathway geometry for a support organ. The end result of the analysis is the identification of a biofabrication process for a fully biological, air–liquid interface that approaches suitable nutrient and gas-exchange properties and guidance for how subsequent iterations could produce a larger scale membrane for a biofabricated booster lung.

## 2 Materials and Methods

### 2.1 Hydrogel Fabrication, Processing, and Storage

To create the thin COLL I sheets, a COLL I solution was cast and polymerized into a hydrogel, dehydrated, and then rehydrated to increase its mechanical strength similar to previous approaches.^34^ Briefly, the COLL I stock solution (Corning Life Sciences, Corning, NY, USA), distilled water (ddH_2_O), 10x phosphate buffered saline (PBS), and 1x NaOH solutions were combined. Then, 0.5 mL of solution was deposited into circular, silicone molds (20 mm inner diameter (ID)) pressed onto glass coverslips. Gelation occurred in a humidified incubator at 37 °C for 30 min, forming a hydrogel with an initial thickness of 1.6 mm (Figure 1(A)). The molds were removed, and the hydrogels soaked in ddH_2_O for 12 h to leach salt. The ddH_2_O was aspirated and each hydrogel was dehydrated in a biohood overnight (Figure 1(B)). The wash-dry steps were repeated for a total of two cycles for a uniformly transparent, salt-free appearance, and the COLL I hydrogels were stored dry until use (Figure 1(C)).

**Figure 1:**
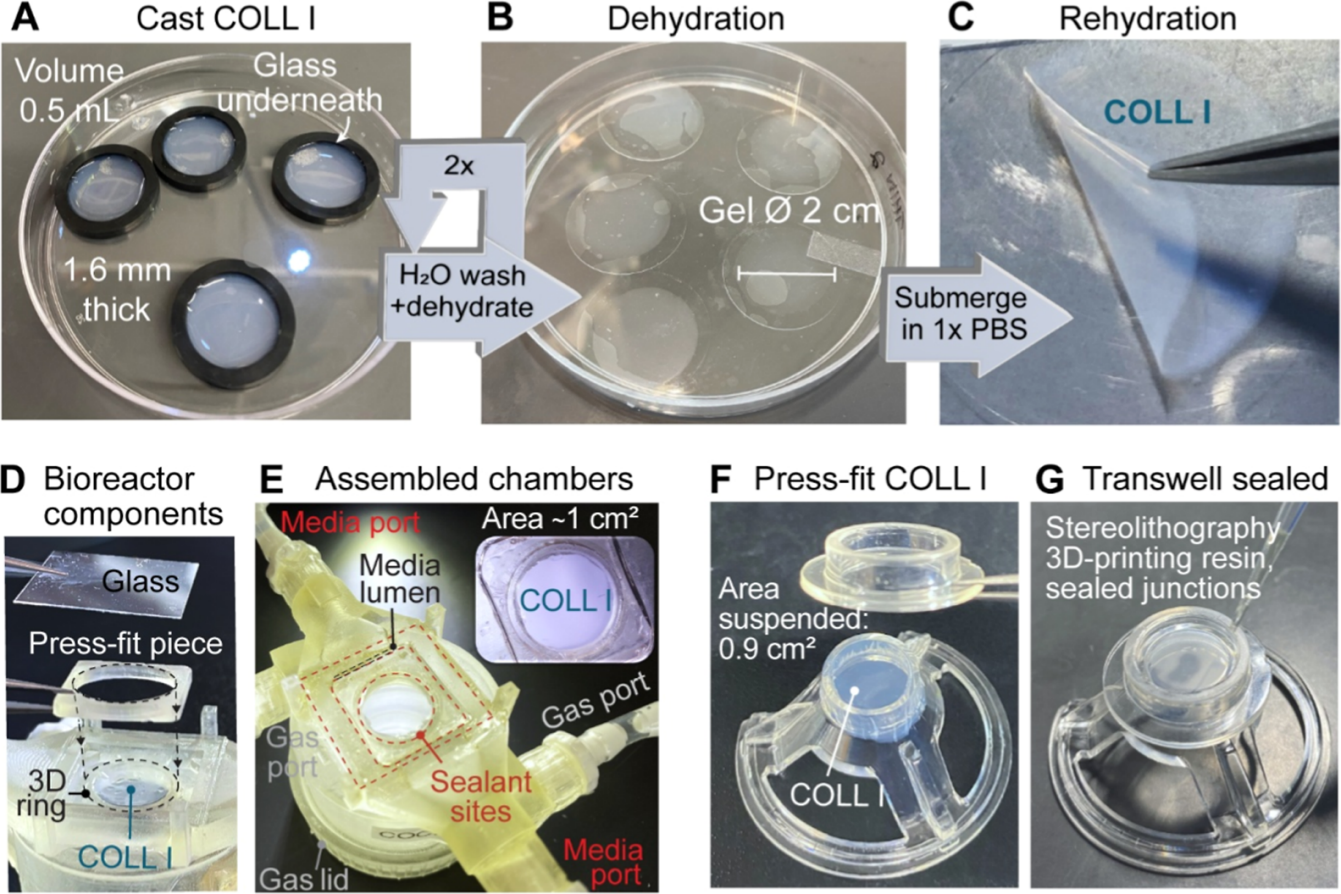
Steps for collagen type I (COLL I) membrane fabrication and incorporation into a gas-exchange bioreactor system or Transwell configuration for characterization. (A) The COLL I solution was cast into molds, and (B) after a water soak and air dehydration, the dried membrane is stuck onto the underlying glass coverslip. (C) Membranes rehydrated in 1x PBS can then be manipulated with forceps. (D) The COLL I membrane is incorporated into the gas-exchange bioreactor by press-fitting the raised ring portion of the bioreactor body with the press-fit frame component. (E) The system is sealed by applying biocompatible, stereolithography 3D-printing resin at the junctions of parts and curing with an ultraviolet light to harden the material. Assembled chambers are separated by ∼1 cm^2^ of the membrane area capable of gas transfer between compartments. More details on bioreactor design and construction are rendered in Supporting Information Figure S1. (F) For cell culture in Transwells, the plastic membrane of a commercial product is replaced with the COLL I membrane alone and (G) junctions again sealed with the cell-safe, 3D-printing resin.

### 2.2 Quantification of Rehydrated Scaffold Thickness

The relationship between the concentration of the COLL I casting solution and the final hydrogel thickness after dehydration–rehydration was first studied to identify a set of parameters that produce thin but mechanically stable gels for subsequent work. COLL I membranes (N = 8/group) were cast with solutions between ∼2 and 6 mg/mL, postprocessed as described, and imaged while submerged in 1x PBS. 3D second harmonic generation image volumes of four regions per sample were acquired with a Nikon A1R MP + Multiphoton Microscope (Nikon, USA) with a 25× objective (λ = 820 nm, 0.5 μm step size). Utilizing a custom ImageJ (NIH) macro, each 3D volume was converted to a mask to identify edges of the hydrogel and imported into MATLAB R2019b (MathWorks) to compute thickness values.^35^ All subsequent experiments utilized a COLL I membrane cast to an initial 1.6 mm-thick hydrogel at a concentration of 3.8 mg/mL. This concentration produced a thin membrane with a final rehydration thickness of 18.8 ± 3.6 μm that could be manipulated easily with forceps without tearing. The stability and reproducibility of this final COLL I hydrogel thickness was evaluated by quantifying the thickness of separate sets (N = 4/group) of COLL I hydrogel samples made under identical conditions that had been soaked in 1x PBS for a total of 0, 24, or 72 h.

### 2.3 Oxygen Transfer across the Acellular, COLL I Membrane

#### 2.3.1 Gas Transfer Bioreactor Design, Construction, and Assembly

To evaluate the oxygen transfer capabilities of the fully biological membrane, a custom bioreactor was made to incorporate and measure oxygen (O_2_) movement across the acellular COLL I membrane (18.8 ± 3.6 μm) into cell culture medium. A medical grade, silicone elastomeric product (51 μm-thick) from BioPlexus (Kingman, USA) was selected and tested as a gold standard, gas-exchange material. This product was one of the thinnest, commercially available sheets of silicone in the thickness range of membranes utilized for synthetic microchannel artificial lungs (∼6–130 μm).^36,37^ Custom bioreactor components consist of the main body, press-fit frame, and lid for the gas space (Figure 1(D)). They were designed in SOLIDWORKS 2021, printed with stereolithography Dental SG resin on a Form 3B printer (Formlabs, USA), and postprocessed according to standard procedures.^38^ Bioreactors with no membrane or a gas impermeable interface were utilized as positive and negative controls. In-depth, gas-exchange bioreactor parameters and dimensions can be found in Figure S1.

Each membrane was incorporated into a bioreactor (N = 6/group) using a press-fit method to suspend 1.07 cm^2^ of area between cell culture media and gas spaces (Figure 1(E)). The COLL I membranes were exposed to 10 min of UV-Ozone in the Novascan PSDP Pro Series chamber (Novascan Technologies, Ames, IA, USA) prior to the press-fit for sterility purposes. The hydrogel was transferred into a reservoir with 1x PBS to keep it unfurled as the bioreactor contacted it from below and lifted it out of the fluid. This motion positioned the hydrogel across the extruded ring of the bioreactor body, and the larger diameter of the membrane caused it to protrude over the ring’s circumferential edge. Slotting the press-fit frame onto the extruded ring put pressure on the overhang region and pulled the COLL I membrane taut in an air-suspended state. For the synthetic polymer group, the silicone elastomer was cut to a diameter of 16.6 mm and sonicated in 100% ethanol for 1 h to clean the material before it was press-fit through identical means. Dental SG resin was used to seal gaps between press-fit pieces for both groups by pipetting it onto sealant sites and curing for 15 min with the Novascan PSDP Pro Series chamber to harden it. A glass coverslip was placed and similarly sealed with resin to close the media chamber, while the gas space became airtight by screwing on the gasketed lid (Figure S1­(E)). Each bioreactor was connected to media and gas pathways using 1/8″ ID tubing and commercial adapters (Advantor, USA) for gas transfer runs.

#### 2.3.2 Oxygen Transfer into Static Cell Culture Media

The oxygen permeability of the COLL I and silicone elastomer membranes was evaluated by measuring the change in oxygen partial pressure (pO_2_) of static cell culture media following membrane exposure to flowing 95% oxygen. Endothelial Cell Growth Medium-2 BulletKit (Lonza Bioscience, Walkersville, MD, USA) was used as a medium and supplemented to 1% with Antibiotic-Antimycotic (Gibco, Thermo Fisher Scientific, USA). The medium was gas equilibrated to 5% CO_2_, 95% air incubator conditions in cell culture flasks with filters for 48 h before use. This step was performed to reduce its proportion of dissolved oxygen and increase the diffusion gradient for O_2_ transfer. On the experiment day, a sterile, airtight, glass syringe (Hamilton, USA) was loaded with the media, capped, and prewarmed to 38 °C. The bioreactor was primed with 1.2 mL of media by perfusing the fluid pathway shown in Figure S1F­(i,ii). A syringe with a 2″ needle was used to aspirate any liquid in tubing distal to the bioreactor to ensure media in the line external to the gas-exchange chamber would not dilute final pO_2_ values. Three baseline medium samples of 300 μL volumes were collected from the glass syringe immediately after priming, and the dissolved pO_2_ was quickly measured in 65 μL aspirations with an ABL800 FLEX blood gas analyzer (Radiometer America, Brea, CA, USA). The bioreactor was insulated on a hot plate to maintain a stable internal temperature of 37 ± 2 °C during gas transfer (Figure S1­(F)). Gas flow at 5 mL/min was provided to the bioreactor’s gas inlet from a 95% O_2_ and 5% CO_2_ tank. The gas flow rate was set with a gas flow controller (MFLX32044-00, Masterflex, Avantor, USA) and measured using a mass flow meter (FMA-1616A, Omega Engineering, Norwalk, CT, USA). After 1 h of gas transfer, 1 mL of the fluid within the bioreactor was aspirated and split into three 1 mL sample syringes for pO_2_ measurements, analogous to the baseline samples.

### 2.4 Acellular Hydrostatic Plasma Permeability

To evaluate the COLL I membrane’s ability to retain pressurized fluid, plasma permeability was measured by leveraging a fluid column of bovine plasma (heparinized, 6.0 g of protein/dL, LAMPIRE, Everett, PA, USA) as a test system. Each membrane (*N* = 7) was sealed into the base of the column inside of an incubator (37 °C) and subjected to ∼15 mmHg for 5 h. This pressure is an applicable, physiological value representative of pressures in the pulmonary artery (PA), and the PA is a potential site for attachment of a biofabricated lung to the vasculature.^31^ The hydrogels were press-fit and sealed into a Dental SG printed, two-part holder to create a 0.89 cm^2^ suspended area. The holder resembles a Transwell but has an X-shaped support to correct membrane curvature under the sustained pressure for a flat, known area. The column was made with 1/4″ ID tubing and connected to the holder using a stopcock and a custom, printed part (Figure S2­(A)). The membrane was hydrated for 10 min before testing. The total change in plasma height was measured with a caliper every hour and the new pressure provided at the start of each hour, given the minor changes in column height, is recorded in Figure S3 for reference. Plasma permeability for each hour interval was calculated as μL/cm^2^/mmHg/h and reported as the average across the time points ± the standard deviation.

### 2.5 Acellular Burst Pressure Testing

The pressure at which the COLL I membrane tears or ruptures and lets liquid through, referred to as burst pressure, was characterized with a fluid column as a metric for mechanical stability.^31,39^ We selected 120 mmHg, a typical systolic arterial blood pressure, as a maximum value for testing because it far surpasses the blood pressure expected in the biofabricated lung if it is attached to the pulmonary artery. Similar to the plasma permeability assay (Figure S2­(A)), the commercial Transwell membrane (3407, Corning Life Sciences) was removed and replaced with the COLL I hydrogel for an area of 0.89 cm^2^ through the methods in Figure 1(F,G). Each membrane (*N* = 7) was subjected to 15 min of UV-Ozone sterilization before incorporation to mimic handling of cellularized scaffolds. The Transwell was sealed into the wide end of a syringe without its plunger using SLA 3D-printing resin and the syringe’s luer connected to a stopcock at the base of the tubing (Figure S2­(B)). PBS (1x) at room temperature was added dropwise and the membrane continuously inspected for instant or progressing rupture. After reaching 120 mmHg, the pressure was held constant for 3 min and further inspected for signs of damage. Samples that survived up to 120 mmHg or never failed were recorded as having burst pressures of 120 mmHg or >120 mmHg, respectively.

### 2.6 Cell Culture

Pooled donor human umbilical vein endothelial cells (HUVECs) (Lonza Bioscience) and A549 (ATCC, Manassas, VA, USA) distal lung epithelial cell lines were expanded according to standard protocols and used in accordance with their ethical guidelines.^40,41^ HUVECs were cultured with endothelial cell growth medium-2 BulletKit media (CC-3162, Lonza Bioscience) supplemented to 1% with Antibiotic-Antimycotic (Thermo Fisher Scientific, USA). A549 cells were cultured with F–12K Media (ATCC, Manassas, VA, USA) supplemented with 10% Fetal Bovine Serum (Sigma-Aldrich, Burlington, MA, USA) and with 1% Antibiotic-Antimycotic. HUVECs and A549s were lifted with 0.25% Trypsin–EDTA with Phenol Red (Gibco, Thermo Fisher Scientific, USA) and spun down, respectively, at 200*g* or 125*g* for 5 min. HUVECs were used at passage six or below.

### 2.7 Transwell Incorporation of the Collagen I and Cellularization

To produce cellular membranes for various characterization experiments, COLL I Transwells were made by replacing the original membrane of commercial Transwells with the COLL I hydrogels and seeding with HUVEC and/or A549 (Figure 1(F,G)). The COLL I Transwells were sterilized beforehand through 20 min of UV–ozone exposure within a NovaScan ProSeries chamber. For coculture groups, the type of characterization assay being conducted on the samples determined which side of the membrane (top/bottom) was appropriate to seed with HUVECs or A549s. Steps for establishing a coculture are shown in Supporting Information Figure S4, and single culture groups utilized the relevant subset of these methods. Cell seeding densities were consistently 50,000 cells/cm^2^ for HUVEC and 75,000 cells/cm^2^ for A549s. Both cell types were allowed to adhere for 3 h in a 5% CO_2_, humidified incubator (37 °C) in their specific media, but afterward, only HUVEC medium was provided. The samples that experienced air–liquid culture conditions always placed the endothelium in direct contact with culture media and the epithelium in contact with air. For comparable humidity levels between coculture and single culture groups, medium was still given to the acellular side of single cell-type membranes when appropriate. The cell suspension on the top of the membrane (250 μL) or on the bottom (3.5 mL) was exchanged with fresh HUVEC media after seeding and each volume subsequently replaced every 24 or 48 h, in turn.

### 2.8 Viability of Air–Liquid Cocultures

A Live/Dead test (L32250, Invitrogen, Thermo Fisher Scientific, USA) was performed on the cocultured COLL I membranes in Transwells to evaluate whether sufficient nutrient diffusion reaches the epithelial cell layer during air–liquid (A–L) conditions. HUVECs were seeded on the bottom and A549 cells on top of the membrane for control and experimental culture conditions (*N* = 5 each). In the control group, both cell layers remained in contact with media (liquid–liquid culture, Liq.–Liq.) for all 7 days, while the medium was removed from the air–liquid group’s epithelial cell side after 3 days. The A549 side of the A–L group was rinsed with PBS at the time it transitioned to A–L culture and subsequently once every 24 h to contribute to the clearance of epithelial waste products. This step was included in the COLL I Transwell culture model in the static setting to better recapitulate the level of fluid clearance present in a perfused booster lung. To test viability on day 7, the staining solution consisted of 1:200 NucBlue (Hoechst 33342) to stain nuclei (Invitrogen, Thermo Fisher Scientific, USA), 2 μM Calcein AM for live cells, and 4 μM Ethidium homodimer-1 for dead cells added to Hanks’ Balanced Salt solution (HBSS) with calcium magnesium (Millipore Sigma, Burlington, MA, USA). Each side of the membrane was incubated with 250 μL for 15 min in the dark at 37 °C. Confocal images were acquired as 3D volumes (3 μm z-step) at four locations per sample. Spot detection in Imaris (version 9.8, Oxford Instruments, USA) was used to obtain the total cell number and count live (green) and dead (red) cells colocalized with nuclei for each culture group.

### 2.9 Cellularized Membrane Permeability to 70 kDa FITC-Dextran

Both the native alveoli and a biofabricated lung can experience fluid imbalances that result from albumin leakage because an abnormal efflux of albumin accelerates water transport into an airspace.^31,42^ Therefore, acellular and cellular COLL I membranes were tested to determine their permeability to an albumin mimic and assess each layer’s contribution to barrier function. Seventy kDa dextran molecules conjugated to fluorescein isothiocyanate (FITC) (Millipore Sigma, Burlington, MA, USA) were utilized as an albumin mimic at a concentration of 20 mg/mL, roughly half the concentration of albumin in blood.^43^ Test groups consisted of acellular, HUVEC-only, A549-only, or cocultured membranes after 7 or 14 days of culture. Transwells were seeded with HUVECs on top and A549 cells on the bottom where applicable and transitioned to A–L conditions after 4 days of Liq.–Liq. culture. The dextran source solution was prepared in colorless, phenol red-free Endothelial Cell Growth Basal Medium (EBM CC-3129, Lonza Bioscience) with EGM-2 supplement packs (CC-4176, Lonza Bioscience). All solutions were warmed to 37 °C, and cell layers were washed with HBSS immediately before testing. Next, 3.5 mL of the media without dextran was added to each well of a six-well plate, and 0.2 mL of the dextran source solution deposited inside of the Transwell. A partial volume exchange of 70 μL out of the 200 μL top source solution (20 mg/mL) was conducted at 30 min intervals. As demonstrated in Supporting Information Figure S5 through preliminary experiments, this technique gives the source concentrations a more consistent, driving force for diffusion over the several hours it takes for sink solutions to fall within the detectable concentration range. Media at the base of the wells was sampled in triplicate after 3 h and read (wavelengths: 492 nm, 518 nm) with the SpectraMax i3x spectrophotometer (Molecular Device, San Jose, CA, USA) at 37 °C. The fluorescence (*X*) of each sample was correlated to its dextran concentration (*Y*) using a standard curve. The standard curve was made using dextran concentrations between 0.001 and 0.5 mg/mL and best fit with a simple linear regression model (*Y* = 9.436 × 10^–10^X). Permeability was calculated by dividing the final sink solution concentration by the starting source concentration, area (cm^2^), and time (hours) using established methods.^33,44,45^

### 2.10 Transwell Membrane Cell Staining, Imaging, and Tissue Thickness Measurements

The cellular membranes from day 7 permeability experiments were fixed and stained to visualize and characterize their tissue morphology. They were stained for nuclei, actin, epithelial or vascular endothelial cadherin junctions (E-CAD or VE-CAD), and tight junction zonula occludens-1 (ZO-1). All antibodies and dyes were purchased from Invitrogen, diluted from their stock in 1x PBS, and incubated overnight at room temperature. Briefly, cellular membranes were rinsed 3x with 1x PBS with calcium and magnesium, fixed for 10 min in 4% paraformaldehyde with 0.1% Triton-X-100, and then washed 3x with 1x PBS for 5 min. They were blocked with 5% v/v goat serum (Gibco, Origin: New Zealand) for 1.5 h and rinsed 3x with 1x PBS for 5 min. Samples were stained for primary and then secondary antibodies in a 200 μL volume/side for sequential 12 h durations. Both cell types were stained with mouse anti-ZO-1 primary antibody (1:80), in combination with rabbit anti-ECAD (1:200) or mouse anti-VE-CAD (1:50) for A549 epithelial cells and human umbilical vein endothelial cells, respectively. Following PBS wash 3x for 5 min each, secondary antibodies were added also containing DAPI (1:200 from 5 mg/mL stock) to see nuclei and Alexa Fluor 633 phalloidin stain (1:60) for actin. The secondary antibodies used were an antimouse AlexaFluor 555 conjugate (1:60) and a goat antirabbit AlexaFluor 488 conjugate (1:60). Following the secondary stain, samples were rinsed, and confocal immunofluorescence images were taken at four random locations per membrane with a 25× objective on the Nikon A1R MP + Multiphoton Microscope (1 μm z-step). Multiphoton images were additionally taken at four locations (λ = 820, 0.5 μm step size) to visualize the collagen layer and cell nuclei.

The fluorescence images were quantified to determine the thickness of individual cell layers for both single and cocultured membranes with a custom ImageJ macro.^32,35^ In ImageJ, the cellular membrane was aligned in 3D space with the XY plane, Z-projected, averaged along the *X*-axis, and then signals combined to create a YZ cross-sectional image that shows all color channels. The resulting 2D, black and white image simultaneously depicted signal from nuclei, cellular junctions, and the cell body to reveal the full cell layer thickness. Images were cropped and saved as separate files showing a single cell type. Each image was converted to a binary matrix with ImageJ’s Auto Local Threshold and type “Phansalkar” and then processed with the Find Edges and Skeletonize commands and imported into MATLAB for thickness calculations.^32,35,46,47^

### 2.11 Method Expansion for Channel Casting

Methods from Section 2.1 were applied to form thin-walled, COLL I channels that maintain an air–liquid interface during perfusion as will be necessary to construct a booster lung. One of several possible geometries, a parallel plate channel structure was selected for construction because it is a common path shape for microchannel artificial lungs and frequently used in mathematical models of gas exchange.^36^ The casting and processing workflow was implemented with the use of a three-piece, custom mold made in SolidWorks and printed on the Ember 3D (Autodesk, San Rafael, CA, USA) with flexible resin, SM-412 (Arkema, King of Prussia, PA, USA) (Figure S6). Similar to the methods of Vernon et al. 2004, paraffin wax was used as a sacrificial material for COLL I casting to provide mechanical support during processing steps and to control the shape of the final lumen.^48^ Mold dimensions set the COLL I hydrogel walls to be 1.45 mm-thick at the time of hydrogel gelation. A COLL I casting solution of 4.1 mg/mL was deposited in the mold around custom flow ports, also called flow adapters, with the sheet of paraffin wax (Sigma-Aldrich, St. Louis, MO, USA) spanning the ports’ lumens. Flow ports were made with the same methods as Section 2.3.1 bioreactors, and dimensions are specified in Supporting Information Figure S7­(A). The paraffin sheet was sliced to a 200 μm thickness with a manual rotary microtome (Leica Biosystems, Wetzlar, Germany) and then cut with a paper stencil and a razor blade to specific dimensions that allow the sheet to fit into the flow adapters’ lumens (Figure S7­(B,C)). After casting, the mold parts were disassembled. The channels were transferred into DI water to leach salts and then suspended in a biosafety cabinet’s airflow for 12 h of dehydration. The paraffin wax was dissolved by perfusing the channel with HistoClear II (Electron Microscopy Sciences, Hatfield, PA, USA) through the flow ports for 15 min at 37 °C with a transfer pipet. The channel was washed 5x with DI water and then Section 2.2 methods were applied to obtain the channel wall thickness.

### 2.12 Statistical Analyses

Data was reported as the average ± the population standard deviation. Calculations and statistical comparisons were performed in Prism (GraphPad, version 9.5.1). The type of test was selected based on the nature of each data set with respect to normality, variance, and sample size, and a *P* value ≤0.05 indicated statistical significance. Graphs were generated in Prism, and one to four asterisks depict *P* values of <0.05, <0.01, <0.001, and <0.0001. The relationship between the COLL I solution casting concentration and hydrogel rehydration thickness was characterized through a linear curve fit. To assess membrane stability, COLL I hydrogel thicknesses for different soak durations were compared with a one-way analysis of variance (ANOVA) with nonrepeated measures, followed by a Tukey multiple comparisons test. The same type of analysis was utilized to assess the O_2_ transfer. For A–L culture viability tests, the average fraction of viable cells for each cell type was compared between Liq.–Liq. and A–L culture conditions with Welch’s *t*-test. A Kruskal–Wallis Test and Dunn’s multiple comparisons test were then performed to compare 70 kDa FITC-dextran permeability values between different types of membranes that were tested on the same culture day.

## 3 Results

### 3.1 Assessment of Collagen I Membrane Thickness, Plasma Permeability, and Burst Pressure

To fabricate a biological, gas-exchange membrane, we optimized COLL I casting and dehydration methods to produce a thin but tough hydrogel. Specifically, it needed to be robust enough to withstand physical manipulation and physiological blood pressures, in turn, for the assembly and perfusion of a tissue-based, booster lung. There was an approximately linear relationship between the COLL I concentration (*X*) of the casting solution and the resulting rehydrated thicknesses (*Y*) (Figure 2(A)). Solutions with concentrations of ∼2–6 mg/mL experienced a 2-orders-of-magnitude decrease in thickness during dehydration, from 1.6 mm down to 9.5–34.8 μms. All three test conditions formed COLL I hydrogels that could be peeled away from the glass, manipulated in liquid, and held in air with grasping instruments. This made it possible to assemble and seal them into Transwell style holders and suggests a level of robustness necessary for constructing fluid pathways in a tissue-based oxygenator. The 9.5 μm hydrogels were more prone to tearing than the thicker hydrogels though, so the 18.8 ± 3.6 μm-thick membrane was selected for all subsequent studies as a compromise between mechanical strength and gas-exchange efficiency. These hydrogels demonstrated consistent thickness values independent of their time soaking in 1x PBS (Figure 2(B)). They resisted failure under elevated liquid pressures, and all samples had a burst pressure ≥120 mmHg, demonstrating sufficient strength to handle blood perfusion. When exposed to ∼15 mmHg of plasma pressure over 5 h, the membranes allowed 2.16 ± 0.5 μL/cm^2^/mmHg/h of fluid movement (Figure S4­(B)), suggesting there would be adequate hydration of cells seeded on the air side.

**Figure 2:**
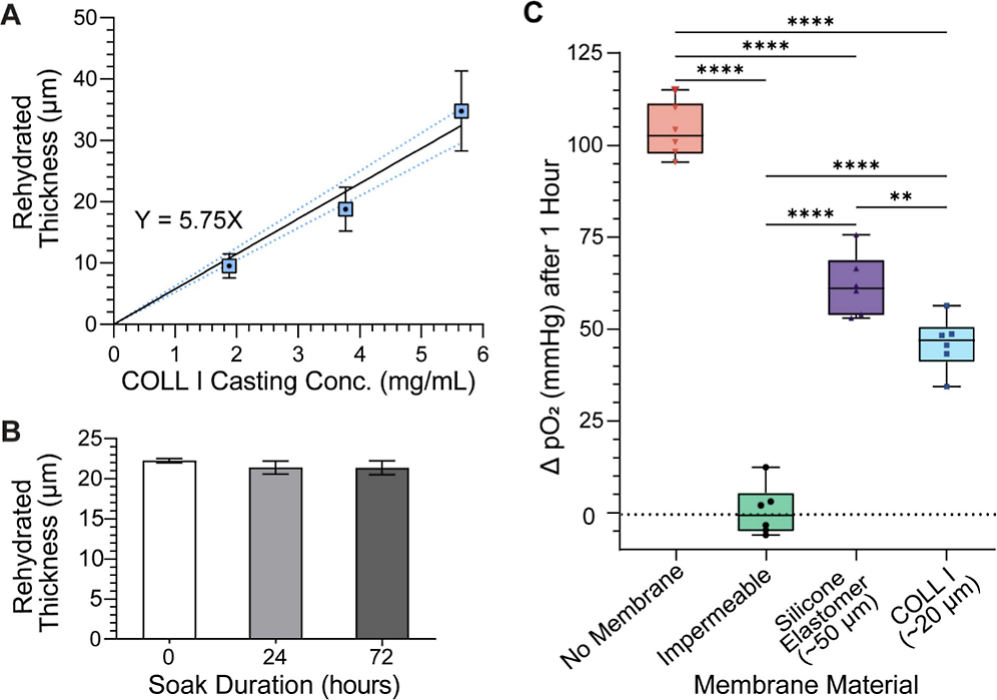
Quantification of COLL I membrane thickness and relative gas transfer capacity. (A) Thickness of rehydrated COLL I membranes vs casting solution concentration with a linear curve (*Y* = 5.75X). (B) Rehydrated thickness vs saline soak duration (One-way ANOVA, *p* = 0.21). (C) O_2_ transfer across COLL I relative to the silicone elastomer and gas impermeable, resin controls.

### 3.2 Acellular COLL I Membrane Oxygen Permeability

Oxygen transfer through the 18.8 μm thick COLL I membrane was evaluated and compared to the no-membrane condition (positive control one), a 51 μm thick silicone elastomer (positive control two), and an impermeable barrier of Dental SG resin several mms thick (negative control). The COLL I membrane group experienced a significant increase in ΔpO_2_ relative to the impermeable barrier (*p* <0.0001) but was less efficient at transferring oxygen than both the silicone elastomer (*p* <0.01) and no membrane (*p* <0.0001) groups, as would be expected (Figure 2(C)). The O_2_ transfer of the COLL I membrane was 74.6% of the silicone elastomer membrane that resembles the gas-exchange interface of synthetic, microchannel artificial lungs.^36,37^ Thus, COLL I membranes have sufficient oxygen permeability for use in a biofabricated lung but will need to be made thinner than highly O_2_ permeable silicone-based membranes to accomplish the same O_2_ transfer (see Discussion).

### 3.3 Viability in Air–Liquid Culture Conditions

Live/dead evaluations of cell layers following air–liquid culture were performed to determine whether enough nutrients diffuse across the endothelium and the ECM hydrogel, in this case COLL I, to support an epithelium in the airspace analogous to the native alveolar membrane (Figure 3(A,B)). Max intensity projection images of the membrane’s surface for day 7 Liq.–Liq. vs A–L coculture conditions (Figure 3(C)) qualitatively show that HUVEC cells (left side) are 99% viable, with no significant differences in viability between Liq.–Liq. and A–L culture (Figure 3(E)). The A549 cell layers (right side) possess a mix of largely alive with some dead cells, with more dead cells counted in A–L (86% viable) than Liq.–Liq. culture (95% viable). Regardless, the vast majority of A549 cells remained alive in A–L after 4 days without direct medium contact, and there was no significant difference between counts. Cross-section views of the coculture locate the dead A549’s to the outermost region of the multilayered cell layer (Figure 3(D)), furthest from nutrients diffusing through the membrane. Thus, even in a static environment, nutrient diffusion across the endothelium and COLL I was sufficient to keep at least a single layer of epithelial cells alive. This shows promise for future work with an induced pluripotent stem cell (iPSC)-derived epithelium because these cells produce a more typical, thin monolayer on the membrane than the A549 cancer cell line in these preliminary studies.^49–51^

**Figure 3:**
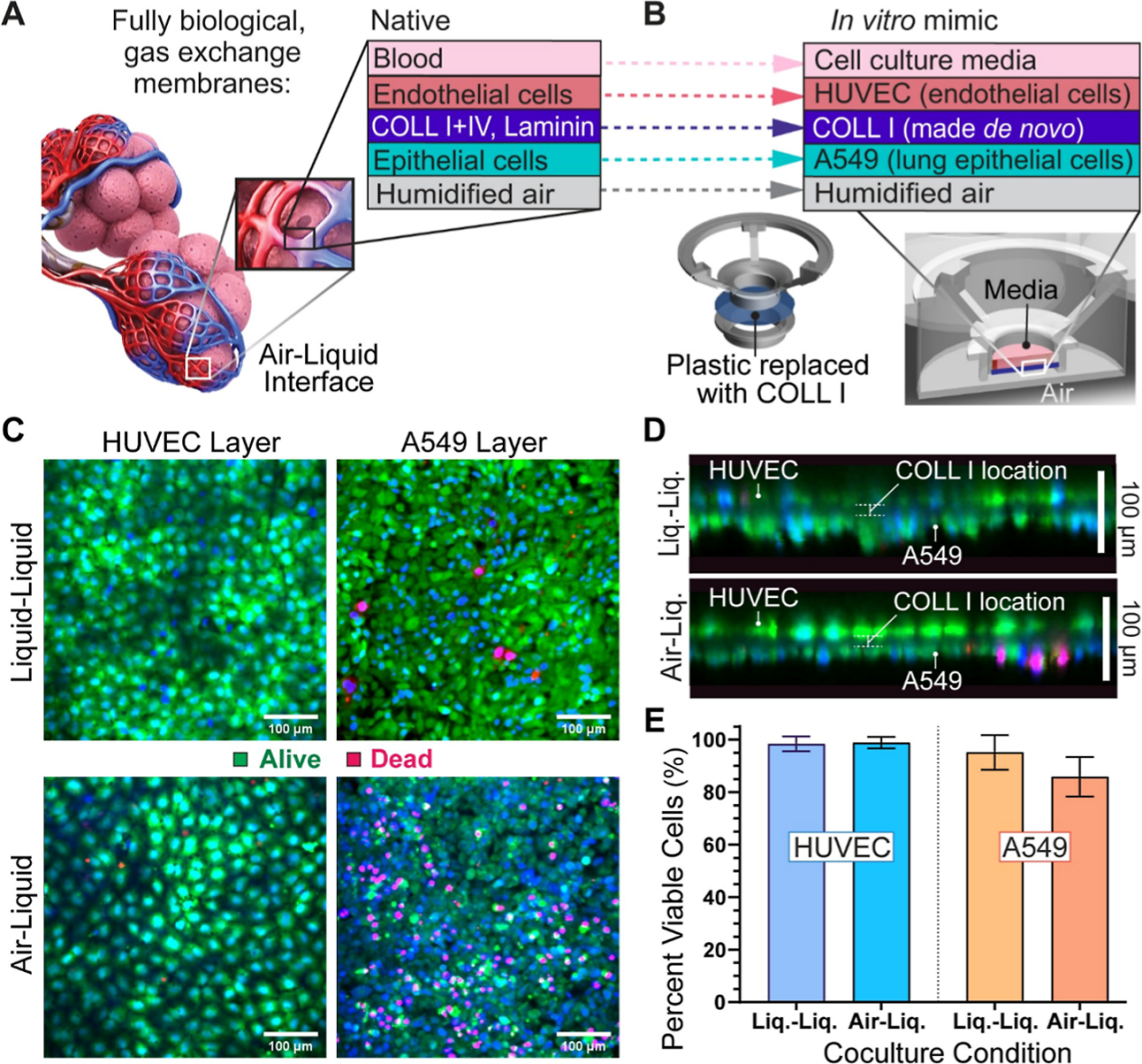
Emulating the native alveolar capillary barrier through the fabrication of a thin, cocultured membrane and evaluating its viability in air–liquid culture conditions. (A) In vivo, two-dimensional gas-exchange membrane.^52^ (B) In vitro membrane with corresponding, biomimetic layers and its incorporation into a Transwell. Regardless of Transwell orientation, the endothelium always contacts media, while the epithelium interfaces with an airspace. (C) Live/dead maximum intensity projections of HUVEC (left) and A549 cells layers (right) in liquid–liquid (top) and air–liquid cocultures (bottom). (D) Representative cross-sectional view for each condition where HUVECs are shown on top and A549 on the bottom of the nonfluorescent COLL I membrane. (E) Percentage of viable cells in each culture condition for the HUVEC layers (Welch’s *t*-test, *p* = 0.785) and A59 layers (Welch’s *t*-test, *p* = 0.072). Abbreviations: liquid–liquid (liq.–liq.); air–liquid (air–liq.).

### 3.4 Tissue Morphology and 70 kDa-FITC Dextran Permeability

To visualize whether HUVECs and A549s formed confluent cell layers that can contribute to barrier function, they were stained for cell–cell junctions and each cell layer was further characterized with respect to morphology and thickness. HUVECs formed vascular endothelial-cadherin junctions (VE-CAD) and A549s formed epithelial cadherin (E-CAD) junctions during A–L culture both as single cultures and a coculture (Figure 4A,B). Both cell types demonstrated the formation of tight junction zonula occludin-1 (ZO-1) at cell edges. The presence of these adherens and tight junctions, as well as the strong actin signal, confirmed the formation of confluent cell sheets with barrier function potential. Confocal immunofluorescence imaging also allowed for morphological characterization and thickness estimations for each cell layer shown in Figure 4C on day 7. Actin staining visualized the full size of the cells in cross-sectional views by generating signal from the cytoplasm and facilitated accurate measures of cell layers’ thicknesses. Single or cocultured membranes shown as cross-sectional views (Figure 4(C,D)) and/or a 3D volume rendering with an additional angled view (Figure 4(C)) indicate HUVECs form a typical monolayer, while A549s transition between a single or multiple cell layer phenotype for all groups. The average thickness of the HUVEC layer was 11.3 ± 1.2 μm for single cell-type cultures and 8.80 ± 1.9 μm for HUVEC cocultures. The thickness of the A549 layer was 33.1 ± 10.4 μm in single culture and 24.08 ± 4.9 μm in cocultures. The cocultured tissues possessed a total membrane thickness of 42.1 ± 7.3 μm. Therefore, the A549 epithelial cell line generated half of the coculture’s overall thickness.

**Figure 4:**
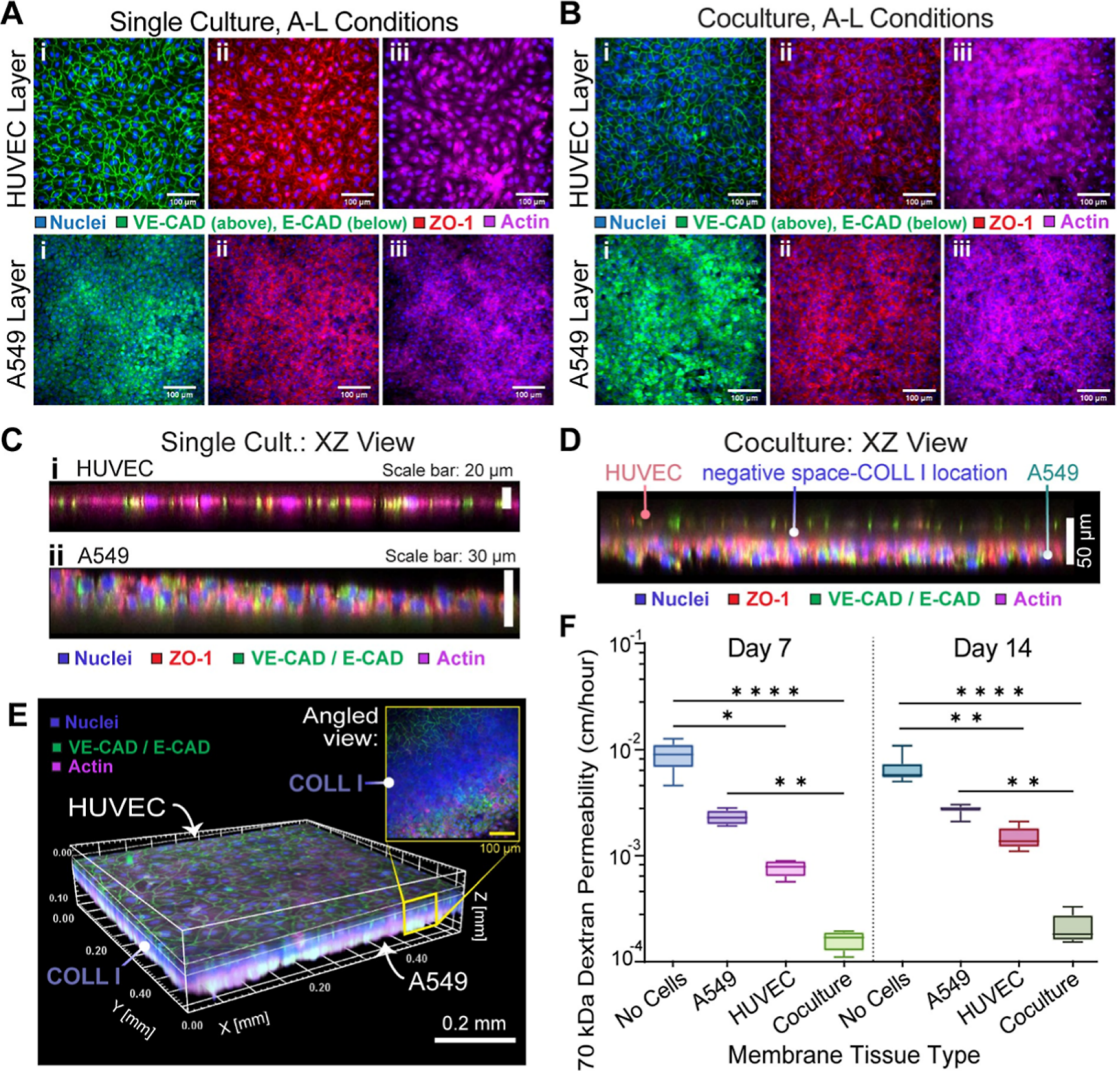
Cellular junction and actin stains for single and coculture tissues on day 7 and 70 kDa FITC dextran tissue permeabilities on culture day 7 or 14. Single culture (A) or coculture (B) max intensity surface projections for the HUVEC layer (top row) and A549 layer (bottom row) showing nuclei with (i) VE-CAD/E-CAD, (ii) ZO-1, or (iii) actin stains. (C,D) Coculture XZ cross-section of confocal images showing the interface thickness for single cultures of (i) HUVEC or (ii) A549s or for the cocultured tissue. (E) 3D volume rendering and angled view of the fully biological cocultured membrane combining confocal cell stains with colocalized, multiphoton images of the COLL I membrane. (F) 70 kDa dextran permeability values for day 7 or day 14 with one-way ANOVA comparisons and data shown as the mean ± standard deviation. Coculture (both days: *p* <0.0001) and HUVEC single culture groups (Day 7: *p* = 0.0135, Day 14: *p* = 0.0058) decreased permeability relative to acellular gels, while A549 single cultures did not produce a significant change (Day 7:0.615, Day 14: *p* = 0.666). Cocultured tissues were comparable to HUVEC single cultures (Day 7: *p* = 0.846, Day 14: *p* = 0.471) and offered better barrier function than A549 single cultures (Day 7: *p* = 0.0061, Day 14: *p* = 0.0059). HUVEC and A549 single cultures exhibited a notable but nonsignificant difference (Day 7: *p* = 0.615, Day 14: *p* = 0.666). The sample sizes for each group consisted of the following: acellular (Day 7: *N* = 6, Day 14: *N* = 8), HUVEC (Day 7: *N* = 6, Day 14: *N* = 8), A549 (Day 7: *N* = 8, Day 14: *N* = 7), or cocultured membranes (Day 7: *N* = 7, Day 14: *N* = 8). Abbreviations: human umbilical vein endothelial cells (HUVECs); vascular endothelial cadherin (VE-CAD); epithelial cadherin (E-CAD); Zonula Occludin-1 (ZO-1).

The functionality of the expressed cell–cell junctions was then quantified with respect to the large molecule permeability of 70 kDa dextran, mimicking serum albumin. For day 7 vs 14 time points, comparisons between tissue types yielded similar trends. The addition of HUVEC to the COLL I hydrogel for a single cell culture decreased dextran permeability when compared to the ECM alone for both time points (Day 7: *p* = 0.0135; Day 14: *p* <0.01) (Figure 4(F)). Combining HUVECs with A549s for a coculture further reduced permeability relative to the acellular control (*p* <0.0001) and relative to the HUVEC single cultures. The latter comparison, however, did not reach statistical significance. A549s in single cultures also saw reduced dextran movement relative to acellular controls (Day 7: *p* < 0.01; Day 14: *p* <0.01) but far worse barrier function relative to the coculture groups (Day 7: *p* = 0.0061, Day 14: *p* <0.01). The A549s possessed slightly worse but nonsignificant differences in barrier function relative to HUVECs. The relative barrier capabilities of epithelial vs endothelial cells in this preliminary membrane therefore contrast the dynamic of the alveolar–capillary interface. In vivo, the type I alveolar epithelial cells that cover 2/3 of the lung’s surface area outperform the barrier function of the semipermeable endothelium in the pulmonary capillaries.^33,53,54^ This emphasizes the need for biofabricated lungs to utilize iPSC-derived alveolar cells as soon as possible.

### 3.5 COLL I Scaffolds in Channel Form Maintain an Air–Liquid Interface

Because a biofabricated booster lung will need multiple perfusable channels to scale-up its gas-exchange surface area, these methods for a 2D membrane were applied to make channels as an initial proof of concept and a foreshadow to future work. Fully biological channels were formed specifically with the Transwell’s target wall thickness and internally perfused while suspended in air. Figure 5 shows how the paraffin functioned as the lumen-shape-specific, supporting material during casting (Figure 5A–D). The introduction of the nontoxic, HistoClear II solution and then DI water for oil-removal allowed for complete wax dissolution, forming an open lumen (Figure 5E,F). The final wall thickness was measured to be 16.8 ± 3.4 μm and therefore approximates other COLL I hydrogel thicknesses in this study. Channels could be manipulated by holding their flow ports with forceps, and they withstood the pressures associated with liquid perfusion with a transfer pipet. When the fluid space was closed off with luer adapters, 1x PBS collected inside and the lumen expanded without any fluid leaking across the walls or from the COLL I–flow adapter interface (Figure 5(G)). Importantly, these compliant, collagen I channels showed no signs of damage during handling steps that could be a part of assembly procedures for a tissue-based, booster lung, and they maintained an air–liquid configuration during perfusion, in alignment with conditions necessary for gas exchange.

**Figure 5:**
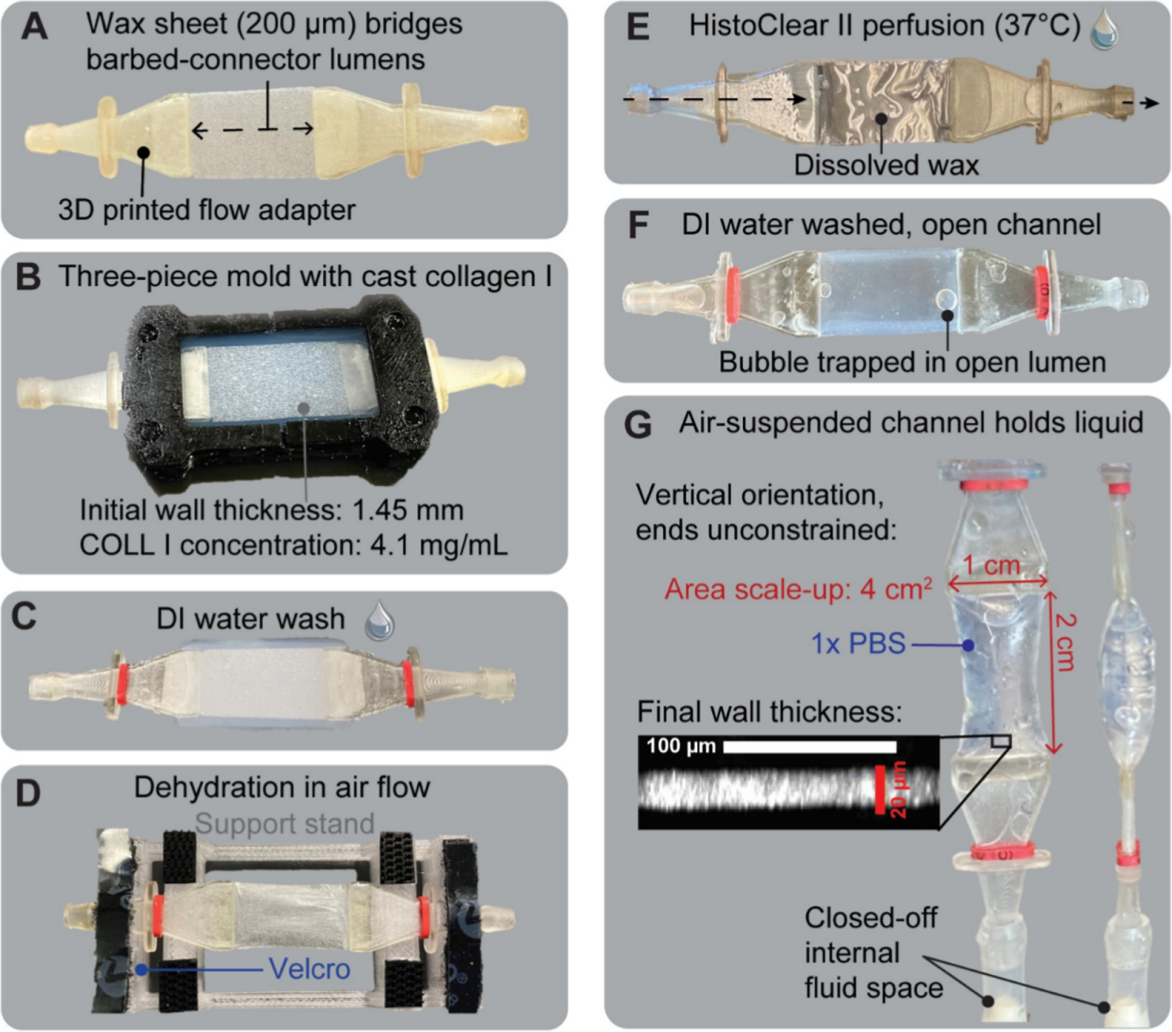
COLL I, thin-walled channels produced by further applying the fabrication approach. (A) A wax sheet and flow adapters, respectively, set the lumen shape and provide flow entry and exit points. (B) Three-piece mold assembly with all negative space filled with the COLL I casting solution. (C) Appearance of COLL I hydrogel postmold release, during the distilled water soak. (D) Appearance of the COLL I hydrogel dehydrated onto the wax and (E) after wax removal through perfusion with HistoClear II. (F) Oil-free, washed channel with an open lumen. (G) Vertically suspended channel holding 1x PBS and providing an air–liquid interface with its wall cross-section shown.

## 4 Discussion

The goal of this study was to fabricate a fully biological membrane and evaluate its potential as a gas-exchange interface of a tissue-based booster lung. To be effective in this role, the membrane must (a) possess adequate mechanical strength to tolerate physical handling and intravascular pressures and (b) transfer oxygen efficiently, so that the full-scale product is a reasonable size for patients. The membrane must (c) be a suitable substrate to maintain viable, confluent endothelial and epithelial layers and (d) exhibit fluid permeability values sufficient for nutrient transport to airway epithelial cells but not so large that liquid exudate floods the airspace.

Collagen I dehydrated–rehydrated membranes with a final thickness of 18.8 ± 3.6 μms were selected for functional analyses due to this group’s mechanical stability, even though production of thinner gels was possible. This thickness proved to be robust enough to be manipulated in liquid and air, sealed into various test devices, and pressurized up to 120 mmHg without bursting.

The collagen I membrane transferred oxygen but not as well as the comparative implantable, medial grade sheet of silicone elastomer from BioPlexus. The change in oxygen partial pressure (Δp_O2_) of the media after 1 h was 61.8 mmHg across the 51 μm-thick silicone elastomer and 46.1 mmHg across the 18.8 ± 3.6 μm-thick COLL I hydrogel. Previous studies have demonstrated that dense ECM membranes with high COLL I content have O_2_ diffusivities ranging from 0.17 × 10^–5^ to 0.61 × 10^–5^ cm^2^/s, while the O_2_ diffusivity of polydimethylsiloxane (PDMS) ranges from 1 × 10^–5^ to 3 × 10^–5^ cm^2^/s.^55–59^ According to Fick’s first law, O_2_ flux scales with the ratio of membrane oxygen diffusivity and interface thickness. Using the ranges of diffusivity values as estimates and considering the comparative thicknesses of the materials, an 18.8 μm-thick COLL I membrane would be expected to have approximately 1/2 of the flux of a 51 μm-thick silicone elastomer. Findings from our study, utilizing an air–liquid test configuration, demonstrate a similar result with the COLL I ΔpO_2_ approximately 3/4ths that of the silicone elastomer membrane. This suggests a reduction in collagen thickness may be required with this approach to make a biofabricated booster lung with surface area requirements in a similar range as silicone-based microchannel artificial lungs.^32,36^ Additional work could focus on strengthening the COLL I to enable the use of thinner hydrogels. One way to do so is through light cross-linking, such as with EDC (1-ethyl-3-(3-(dimethylamino)­propyl-carbodiimide hydrochloride) and NHS (*N*-hydroxy-succinimide). This method conserves free amine groups for cell binding while increasing mechanical strength to three times that of non-cross-linked gels.^60^

The COLL I membrane enabled sufficient nutrient and water transport to support an air–liquid coculture with endothelial cells on the liquid side and epithelial cells on the air side. After 14 days in culture, 10 days in air–liquid conditions, some dead epithelial cells were present on the membrane, but they were largely on the outermost layer of the two to three layers of A549 cells. Thus, a membrane with an equivalent or lesser thickness should be able to support monolayers of primary or induced pluripotent stem cell (iPSC)-derived epithelial cells on one side of the membrane with an endothelial layer on the other. Medium contact on one side was enough to keep the COLL I membrane hydrated. At transmembrane pressures relevant for a booster lung, the acellular COLL I membrane exhibited a filtration rate of 2.16 ± 0.5 μL/cm^2^/mmHg/h. This amounts to 7.8 L/m^2^/day for an acellular membrane exposed to an average pulmonary arterial pressure of 15 mmHg. Typical biofabricated booster lungs will have surface areas on the order of a few square meters, so much like the native alveolus, water will accumulate in the airspace without the endothelial and epithelial layers.^36^ Once cellularized, the membrane filtration rate should reduce this value markedly, avoiding airspace flooding.

A key part of fluid balance, these membranes must restrict the transport of large proteins such as albumin from blood to the airspace to maintain a low, airspace-oncotic pressure. Cellularization of the COLL I membrane with the HUVEC and A549 coculture significantly reduced 70 kDa dextran permeability on day 14 from 6.53 × 10^–3^ to 2.11 × 10^–4^ cm/h. This value exceeds the reported values for albumin permeability across the alveolar capillary barrier for in vitro and in vivo whole lung, animal studies.^53,54^ Goetzman and Visscher 1969, for example, report an albumin permeability of 1.9 × 10^–9^ cm/s equivalent to 7 × 10^–6^ cm/h in explanted canine lungs using an albumin tracer moving from a fluid alveolus into the pulmonary capillaries. This 30-fold difference emphasizes the future need for not just any cells but cell types that more effectively restrict large molecule movement for oncotic fluid balance.^61^

Although useful for initial tests of membrane function, the HUVEC and A549 cells used in this study will need to be replaced with patient specific primary cells or iPSCs as biofabricated lungs progress. Currently, type I and II airway epithelial cells can be generated from iPSCs from patients’ fibroblasts.^62–65^ However, cell culture methods to reprogram and differentiate both are complicated, multiweek processes with low cell yields. Human iPSC endothelial cells can be generated at present but are a heterogeneous mixture of arterial, venous, and lymphatic phenotypes that are likely to have variable responses to biochemical and mechanical cues.^66^ Until iPSC methodologies become more efficient, affordable, and uniform, the research and development of biofabricated lungs should focus on identifying a combination of primary cells that improve the functionality of this type of gas-exchange interface.

This biofabrication approach was additionally utilized to make parallel plate, collagen I channels with a 16.8 ± 3.4 μm wall thickness. These channels demonstrate sufficient mechanical strength to maintain an air–liquid interface in a static configuration and during perfusion without bursting or tearing. These leak-free, proof-of-concept channels represent one type of channel geometry that could be incorporated into designs for full-scale, support lungs.

## 5 Conclusions

A fully biological membrane was fabricated that maintains a coculture of cells in an air–liquid interface out to 14 days. Mechanical stability was achieved using an 18.8 ± 3.6 μm-thick, acellular COLL I membrane fabricated through dehydration methods, and it withstood systemic arterial burst pressures. The acellular COLL I membrane facilitated oxygen transfer, albeit at lesser rates than the silicone elastomer positive control. Future work should examine light-cross-linking of the membranes to improve the mechanical strength and thus enable the use of thinner membranes with more efficient gas exchange. The cocultured COLL I membrane experienced sufficient nutrient transport to support at least one layer of epithelial cells in air–liquid culture conditions. Both endothelial and epithelial cell layers will be necessary in a biofabricated lung to reduce water filtration and albumin permeability values so that they approximate the values of the native alveoli and prevent airspace flooding. Lastly, this biofabrication approach can produce parallel plate, perfusable collagen I channels that sustain an air–liquid interface. Channels like these have the potential to one day function as the assembly unit for an endothelialized, biofabricated, booster lung.

## Supplementary Material



## Data Availability

Data that supports the findings of these studies and developed 3D models are available upon request from the corresponding author.

## Notes

Gas-exchange bioreactor design, assembly, and experimental configuration; hydrostatic plasma permeability vs burst pressure test set-ups; volume and transmembrane pressure data and the resulting plasma permeability values for acellular collagen I membranes in an air–liquid, 37 °C test environment; schematic of Transwell cell seeding steps for a single-culture or cocultured COLL I membrane; source and sink concentration data with a partial volume replacement approach from preliminary, 70 kDa-FITC dextran permeability evaluations with the day 7 acellular or cellularized biologic; collagen I channel casting mold dimensions; and dimensions for the flow adapters and stencil-cut, paraffin wax that allow the wax to slot into the adapters’ lumens (PDF)
