# Supplementary material for: A Fully Biological Gas-Exchange Membrane toward a Biofabricated, Booster Lung
Source: ACS Biomater Sci Eng. 2026 Apr 13;12(5):2746–57. doi: 10.1021/acsbiomaterials.6c00046 (PMC13169300; doi:10.1021/acsbiomaterials.6c00046)
Supplement: Supplementary file 1 [file ab6c00046_si_001.pdf]

Supporting Information

A Fully Biological Gas-Exchange Membrane toward a Biofabricated, Booster Lung

*Erica M. Comber[1, *], Kalliope G. Roberts[1], Isabel M. Joyce[1,3], Rachelle N. Palchesko[1], Daniel J. Shiwarksi[1,3], Xi Ren[1], Adam W. Feinberg[1,2], Keith E. Cook[1]*

[1] Department of Biomedical Engineering, Carnegie Mellon University, Pittsburgh, PA 15213, United States of America

[2] Department of Materials Science & Engineering, Carnegie Mellon University, Pittsburgh, PA 15213, United States of America

[3] Department of Bioengineering, University of Pittsburgh, Pittsburgh, PA 15213, United States of America

* Author to whom any correspondence should be addressed.

Email: ericamcomber@gmail.com

7 Supplemental Figures

- Figure S1: Gas exchange bioreactor design, assembly, and experimental configuration. (PDF)
- Figure S2: Hydrostatic plasma permeability vs. burst pressure test set-ups. (PDF)
- Figure S3: Volume and transmembrane pressure data and the resulting plasma permeability values for acellular collagen I membranes in an air-liquid, 37ºC test environment. (PDF)
- Figure S4: Schematic of Transwell cell seeding steps for a single-culture or cocultured COLL I membrane. (PDF)
- Figure S5: Source and sink concentration data with a partial volume replacement approach from preliminary, 70 kDa-FITC dextran permeability evaluations with the day 7 acellular or cellularized biologic. (PDF)
- Figure S6: Collagen I channel casting mold dimensions. (PDF)
- Figure S7: Dimensions for the flow adapters and stencil-cut, paraffin wax that allow the wax to slot into the adapters' lumens. (PDF)

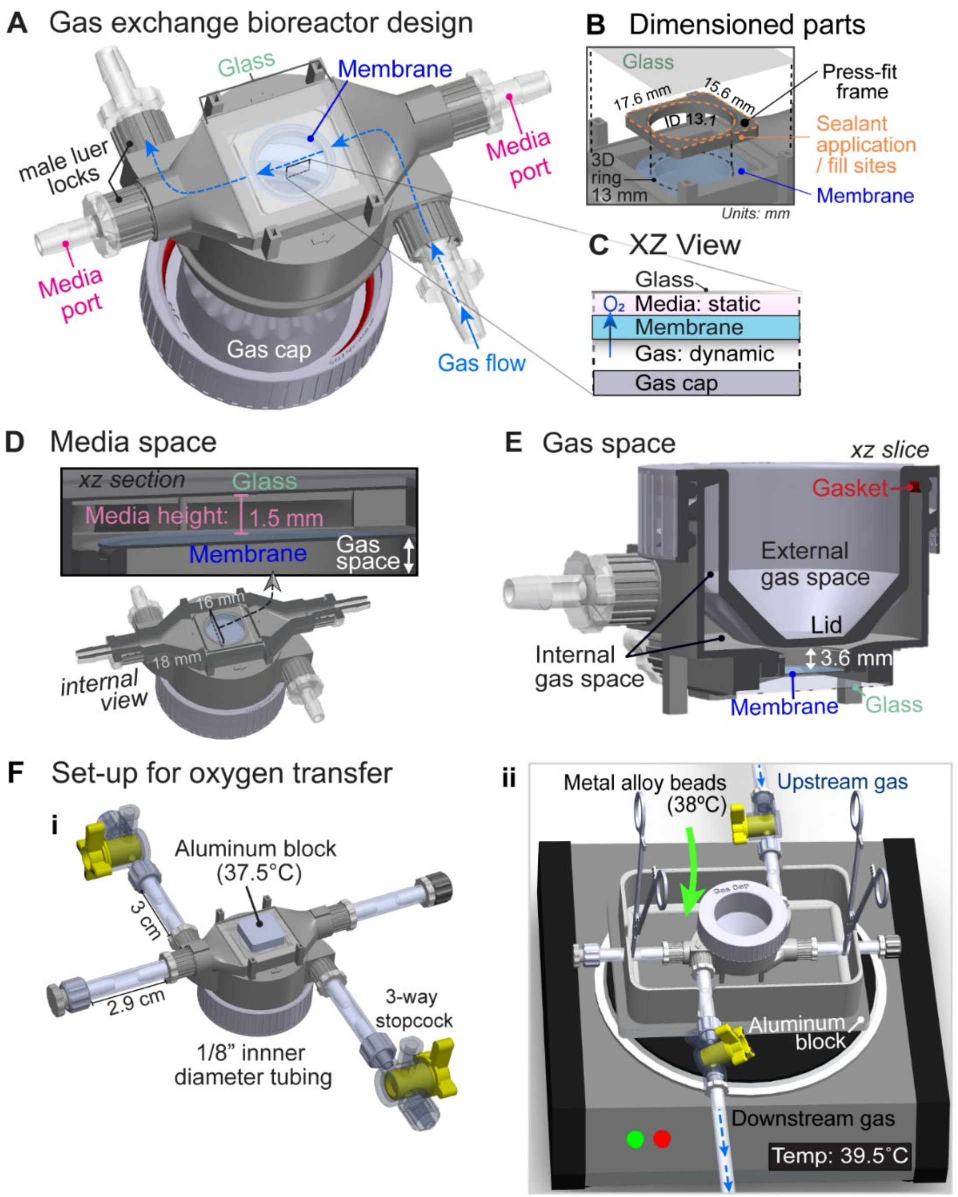

Figure S1. Gas exchange bioreactor design, assembly, and experiment configuration. (A) Bioreactor design with gas flow directed across the membrane and the (B) assembly components involved.(C) Schematic showing that the membrane alone separated media and gas sides. (D) Views of the bioreactor's internal fluid compartment and the XZ cross-section of the parallel plate region. (E) Sectioned view of the gas comparment with the lid present to visualize internal vs external gas spaces. (Fi) This tubing and adapter configuration allowed for entry and exit of liquid and gases, and an aluminum block minimized temperature losses. (Fii) A hot plate generated heat and aluminum beads provided insulation to maintain the static media at 37 ± 2ºC.

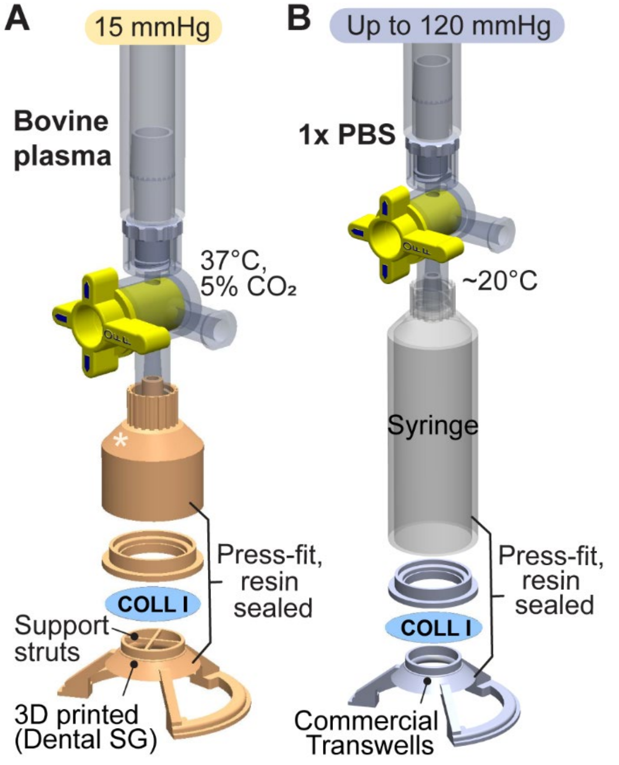

Figure S2. (A) Hydrostatic plasma permeability and (B) burst pressure testing configurations with emphasis on the differences between the set-ups.

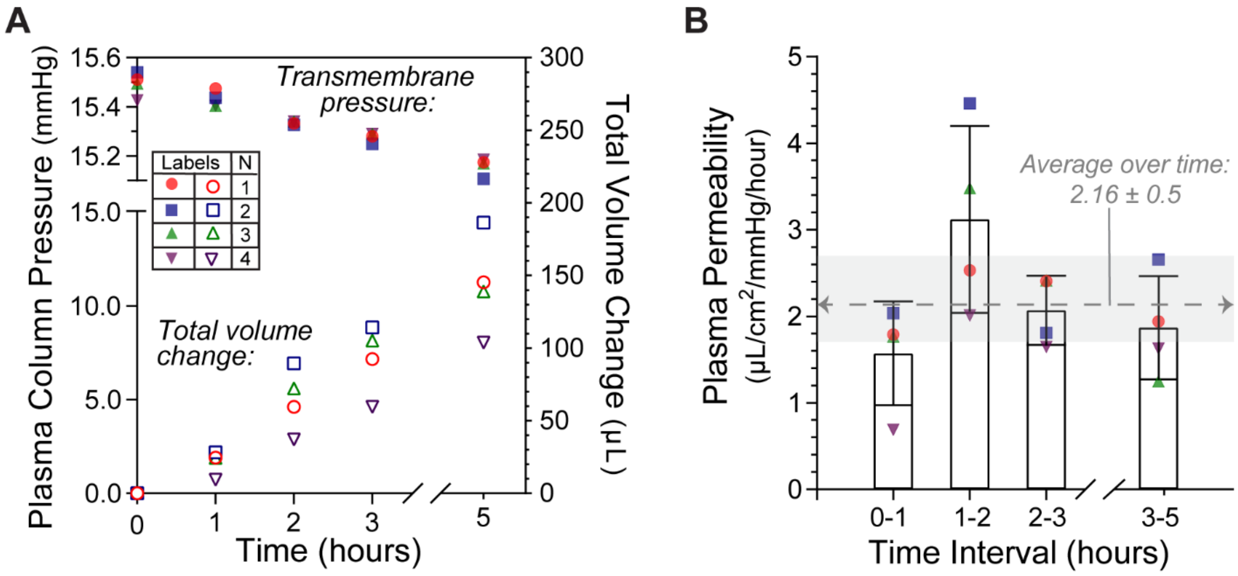

Figure S3. Volume and transmembrane pressure data and the resulting plasma permeability values for the acellular collagen I membranes in an air-liquid, 37ºC test environment (N= 4 samples). (A) Total volume changes from hour 0 until the specified time point based on changes in column height (right Y-axis) and the transmembrane pressure at the start of each hour interval (left Y-axis) (B) Average plasma permeability for each time point and the overall average ± standard deviation across time points.

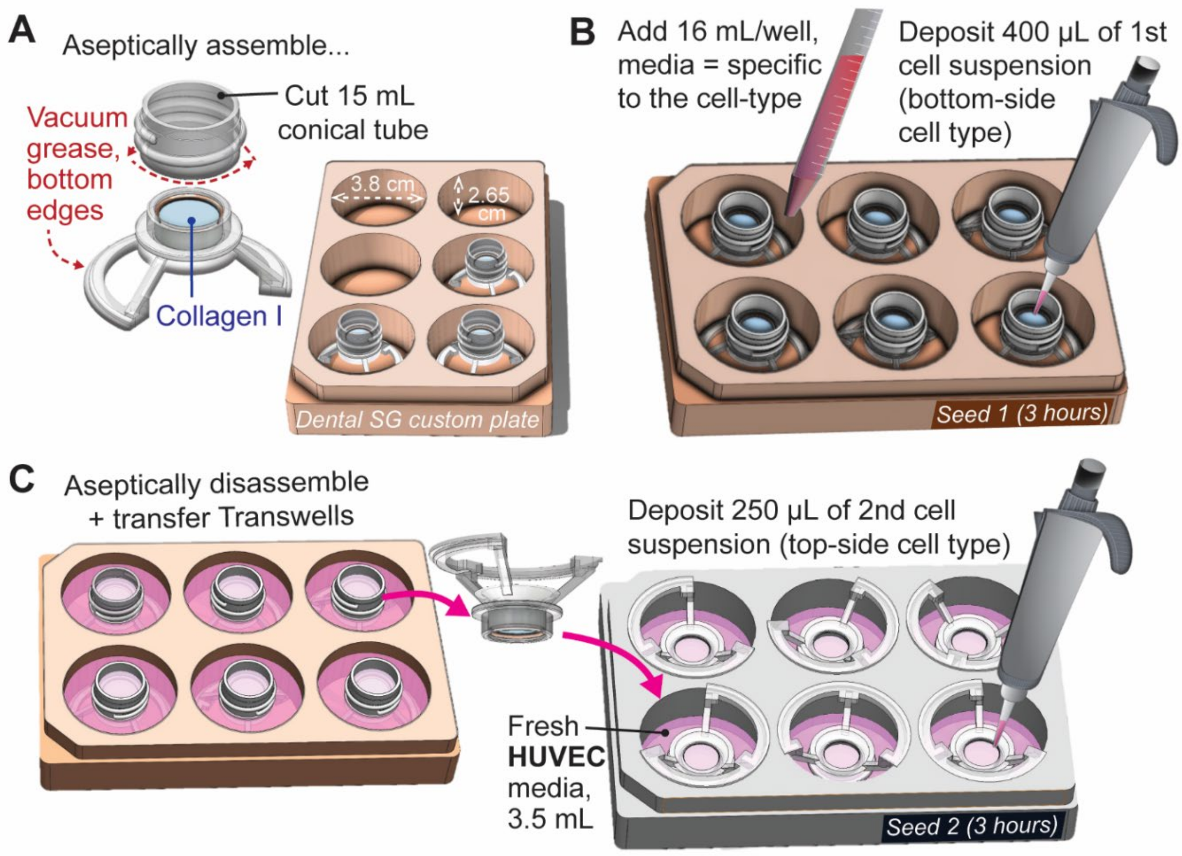

Figure S4. Transwell cell seeding steps consisted of the following: (A) Aseptic assembly of the collagen I Transwells, cut pieces of 15 mL conical tubes acting as temporary containers, and the custom six-well plate. (B) Deposition of media underneath the membrane to minimize its curvature and then seeding of the first cell-type solution within the cut conical on the bottom-side of the membrane. (C) Transwell transfer to a commercial six-well plate and seeding of the second cell type on the top side of the membrane.

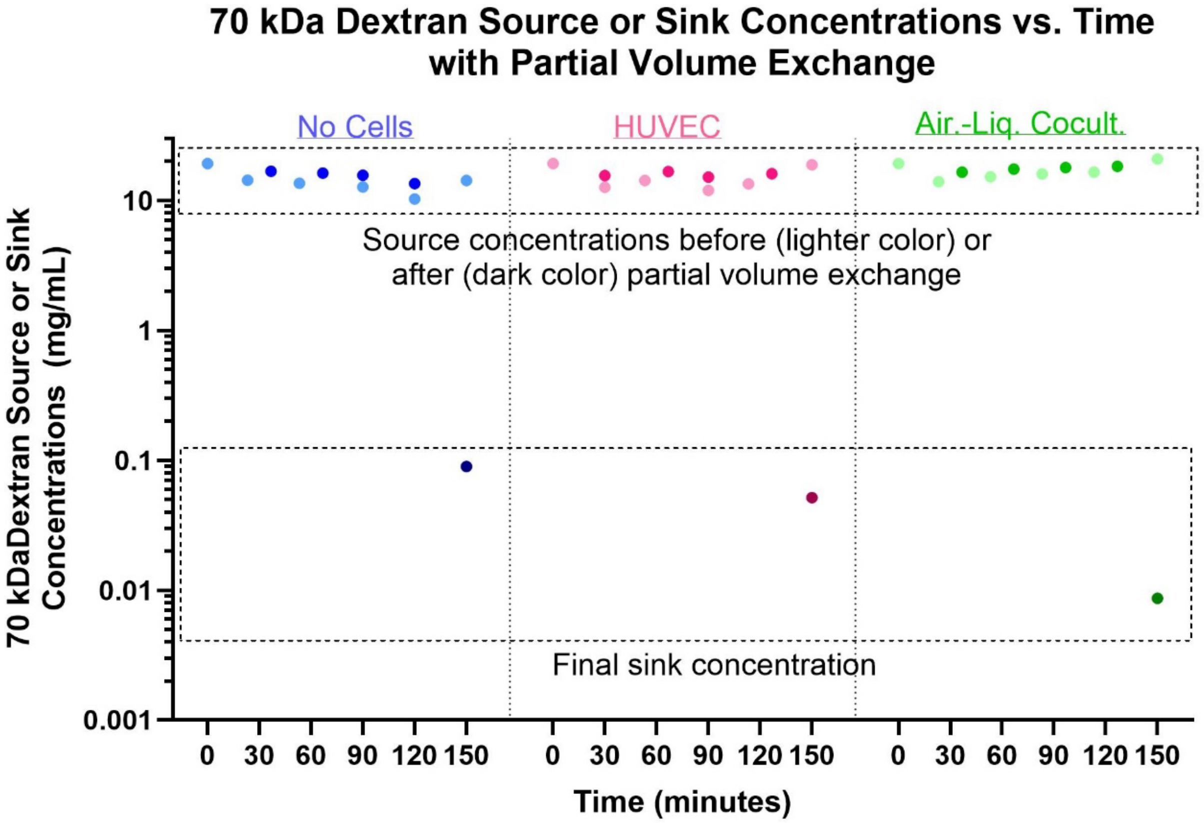

Figure S5. Source and sink 70 kDa-FITC dextran concentration data with a partial volume replacement approach from preliminary permeability evaluations. Test groups include the COLL I membranes with no cells (left), HUVECs only (middle) or a HUVEC and A549 coculture (right), all on day 7 (N=6 per group). Methods for tissue preparation, culture, and assay execution align with section 2.9, except for the use of a slightly shorter, 2.5-hour test duration. Source concentrations are depicted in the top box where the lighter colored markers show the measured top concentration before the partial volume exchange. The dark markers denote the calculated estimate of the source concentration following the exchange. The bottom box's markers signify the final sink concentrations for each group measured at the end of the assay. The range of the Y-axis in $\log_{10}$ starts at 0.001 mg/mL, which was the lowest value detected with precision and accuracy by the spectrophotometer.

A

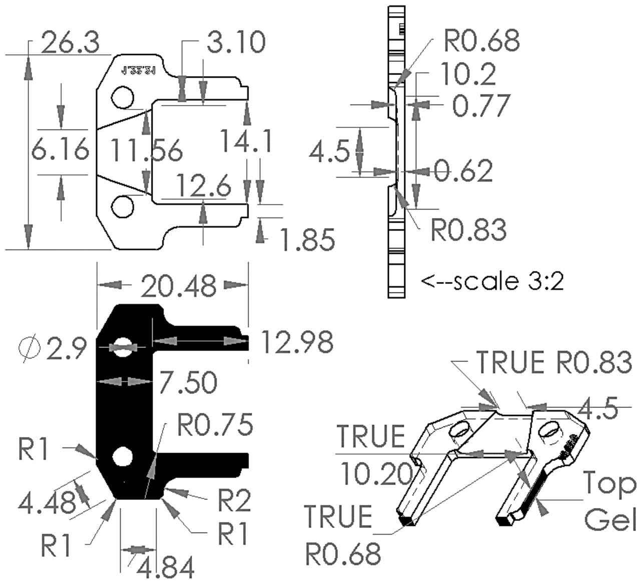

Mold Top for Channel Casting

File: ECOMBER_TOP_MOLDfor10.18.20ADAPTER_20umWALL
Units: mm
Material: SM412-Specialty
Printer: Autodesk Ember
Part: channel casting mold TOP,
1.45 mm gel top and bottom
~1.45 mm side wall
Used 4.1 mg/mL casting solution
Results:~20 um thick top wall

B

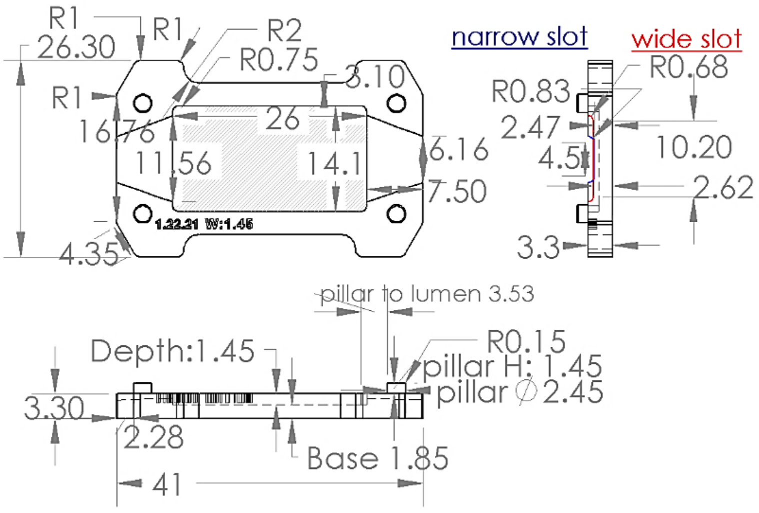

File: ECOMBER_BOTTOM_MOLDfor10.18.20ADAPTER_20umWALL
Units: mm
Material: SM412-Specialty, (CPS)
Printer: Autodesk Ember
Part: channel casting mold bottom,
1.45 mm gel top, bottom, side wall
Used 4.1 mg/mL casting solution
Results:~20 um bottom wall

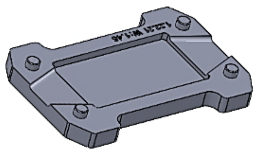

Figure S6. Channel casting part dimensions: design and dimensions for the (A) top and (B) bottom mold components used to cast channels with an initial wall 1.45 mm.

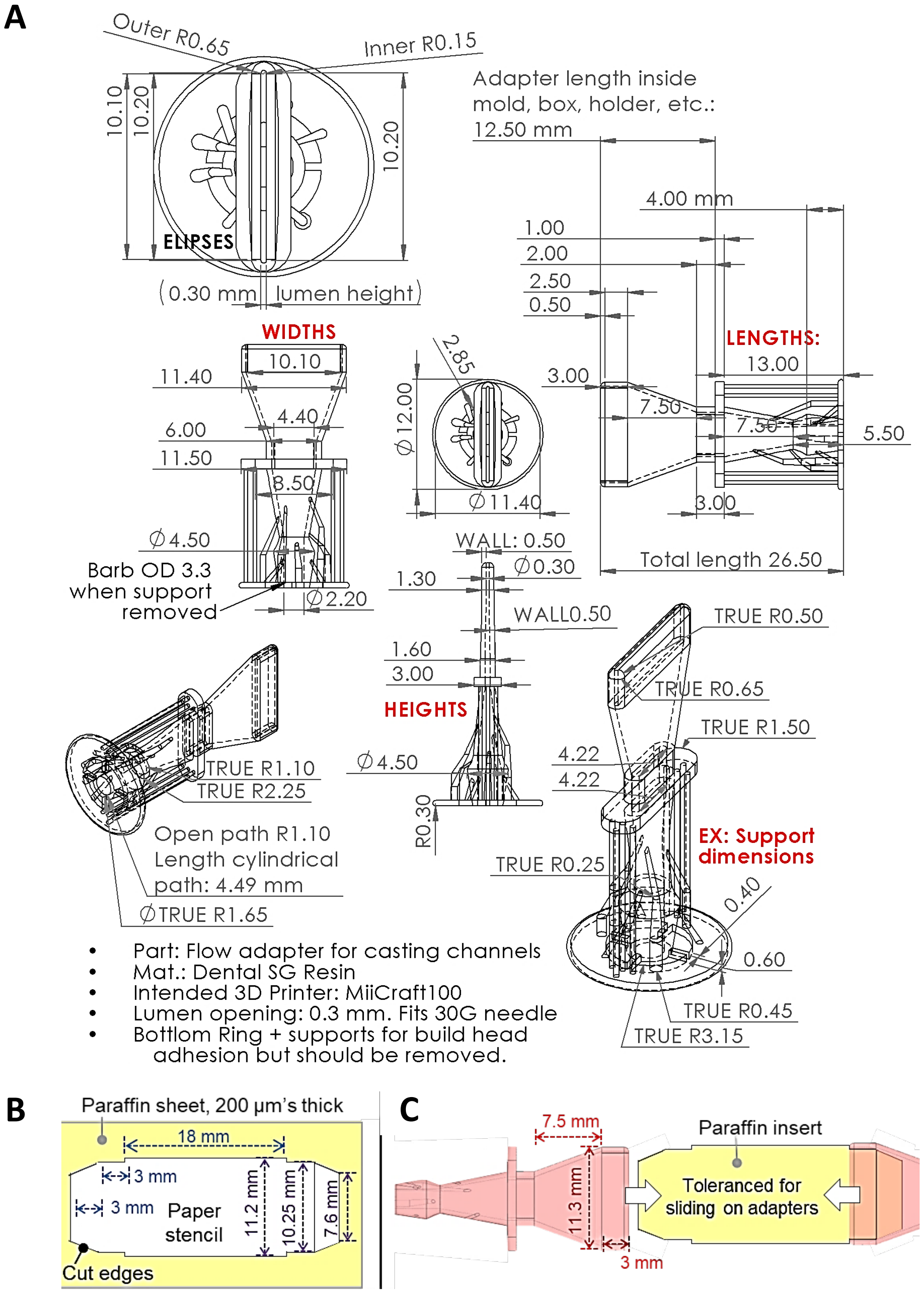

Figure S7. Dimensions for the flow adapters and stencil-cut, paraffin wax that allow the wax to slot into the adapters' lumens. (A) Flow adapter dimensions, specifically selected to allow for fluid transition from a cylindrical to parallel plate geometry. (B) Paper stencil dimensions for cutting a geometrically specific paraffin insert. (C) Pairing of the paraffin wax with the adapters.
